# Demethylation of MicroRNA-124a Genes Attenuated Proliferation of Rheumatoid Arthritis Derived Fibroblast-Like Synoviocytes and Synthesis of Tumor Necrosis Factor-α

**DOI:** 10.1371/journal.pone.0164207

**Published:** 2016-11-08

**Authors:** Qiao Zhou, Li Long, Ting Zhou, Juan Tian, Bin Zhou

**Affiliations:** Department of Rheumatology & Immunology, Sichuan Academy of Medical Sciences & Sichuan Provincial People's Hospital, 1st Ring Rd, Chengdu, Sichuan, 610072, China; Northwestern University Feinberg School of Medicine, UNITED STATES

## Abstract

**Objective:**

To examine the impact of 5-Aza-2ʹ-deoxycytidine (5-AzadC) on methylation status of miR-124a genes in rheumatoid arthritis (RA) associated fibroblast-like synoviocytes (FLS) and its effect on RA-FLS proliferation and TNF-α expression.

**Materials and Methods:**

FLS were isolated from seven RA-derived synovial tissues and cultured in vitro. The expression of miR-124a was measured by real time quantitative polymerase chain reaction (PCR) in FLS with or without 5-AzadC treatment. MiR-124a gene methylation was detected by methylation-specific PCR. FLS were divided into three groups as control, IL-1β and IL-1β/5-AzadC, respectively. The cells in the IL-1β group were treated with 5 μg/L of IL-1β for 24 hours, whereas the cells in the IL-1β/5-AzadC group were first treated with IL-1β exactly as those in the IL-1β group for 24 h but further treated with 1μM 5-AzadC for additional 3 days. The cell growth was estimated based on absorbance at UV450nm. Secreted TNF-α from the cells was evaluated by enzyme-linked immunosorbent assay. After that, RA-FLS treated with IL-1β plus 5-AzadC were further transfected with miR-124a inhibitor or scrambled control. After culturing for 3 days, cell growth and TNF-α concentrations were measured.

**Results:**

After 5-AzadC treatment, the expression of miR-124a was significantly increased compared with the control group (1.545 ± 0.189 vs 0.836 ± 0.166, *p* = 0.001). On the other hand, 5-AzadC significantly reduced IL-1β-mediated cell proliferation by nearly 2.5 fold (*p* = 0.006). Also, the level of TNF-α secreted from the cells treated with IL-1β plus 5-AzadC was considerably less than that from the cells treated with IL-1β alone (324.99 ± 22.73 ng/L vs 387.91 ± 58.51 ng/L, p = 0.022). After transfection with miR-124a inhibitor in RA-FLS treated with IL-1β plus 5-AzadC, the cell proliferation was increased by 18.2% and the TNF-α expression was increased by 19.0% (p = 0.001 and 0.011, respectively).

**Conclusion:**

Methylation of miR-124a genes contributed to IL-1β-mediated RA-FLS proliferation and TNF-α expression.

## Introduction

Rheumatoid arthritis (RA) is an autoimmune disease primarily affecting joints. The characteristics of RA include chronic inflammation, synovium hyperplasia, lymphocyte infiltration and abnormal proliferation of fibroblast-like synoviocytes (FLS), all of which may eventually lead to progressive cartilage erosions and bone destructions [1]. Although the pathogenesis of RA remains largely unknown, genetics and epigenetics may play an important role in RA progression. In particular, recent evidence indicates that DNA methylation, a well-described epigenetic manifestation for many human diseases [2], also occurs in certain autoimmune diseases [3]. However, it is less clear about the specific genes whose methylation are implicated in RA progression. We have recently demonstrated that several regions in the miR-124a genes, which encode a non-coding small RNA with an activity to inhibit cell proliferations [4], were hypermethylated in RA-associated FLS (RA-FLS) [5]. Transfection of miR-124a precursor into RA-FLS also suppressed cellular proliferation and arrested cells at the G1 phase of the cell cycle [6]. Yet, the functional relationship between methylation of miR-124a genes and RA progression has not been established. Because the aggressive growth of RA-FLS contributes greatly to the joint damages [7], our assumption is that demethylation of miR-124a genes would reduce the growth of RA-FLS, thereby reducing RA-associated inflammations. In this study we applied 5-Aza-2′-deoxycytidine (5-AzadC), which has a high potency to inhibit methylation and has been used to treat hematological malignancies [8], to RA-FLS and then examined its impact on the growth of RA-FLS and the expression of inflammatory cytokine tumor necrosis factor-α (TNF-α).

## Materials and Methods

### Isolation and culture of FLS

Synovial tissues were obtained from seven RA patients who had joint surgeries during the period from October 2012 to April 2013 in Sichuan Provincial People’s Hospital. The diagnosis of RA was determined according to the American College of Rheumatology 1987 revised criteria [9]. All patients had signed informed consent forms before their donation of tissues. The patients were numbered from 1 to 7 according to the sequence of their surgeries. The study was performed under an institutionally approved protocol that was in accordance with the Declaration of Helsinki Ethical Principles for Medical Research Involving Human Subjects. The study was also approved by the Ethic Committee of Sichuan Provincial People’s Hospital.

To isolate FLS, synovial tissue specimens were minced and digested with dispase at 37°C for 60 minutes as previously described. After washing, cells were grown in high glucose-containing Dulbecco‘s modified Eagle's medium (DMEM) supplemented with 15% (v/v) heat-inactivated fetal bovine serum (FBS) (Hyclone, USA), 100 U/mL of penicillin (Beyotime, China) and 100 mg/mL of streptomycin (Beyotime, China). Cell cultures were maintained in an incubator of 37°C, 95% humility and an atmosphere of 5% CO2. All FLS between passages 4 and 6 were subjected to experimental procedures.

### Analysis of the expression of miR-124a

Cells were added with 1 μM 5-AzadC (Sigma-Aldrich, USA) and cultured for 3 days. Total RNA was extracted using TRIzol reagent (Invitrogen, USA) from the cells treated with 1 μM 5-AzadC or without. MiR-124a mRNA was detected by real time quantitative PCR using the SYBR Green miRNA assay (Hairpin-it miRNAs Real-Time PCR Quantitation Kit, GenePharma Ltd., China). U6 snRNA was used as the endogenous control for data normalization. The relative miRNA expression was calculated by using the 2−ΔΔCt method. The methylation status in the promoters for three miR-124a genes was analyzed by methylation-specific PCR (MSP) as described previously [5].

### Cell proliferation and cytokine expression

RA-FLS cells were seeded at 2×10^5^/well in 6-well cell culture plates. Cell samples from each patient were divided into 3 groups as control, IL-1β and IL-1β/5-AzadC, respectively, with each group of three wells. The cells of the IL-1β group were treated with 5 ng/mL of IL-1β for 4 days, whereas the cells of the IL-1β/5-AzadC group were first treated with the same concentration of IL-1β and then added with 1 μM 5-AzadC after 24 h, and were continually incubated for 3 days. In contrast, the control cells were incubated in the growth media only for 4 days. At the end of the experiment, the number of cells in each group was estimated based on absorbance at UV_450nm_ using BIO-RAD550 enzyme-labeled instrument. The percentage of cell growth was calculated based on the formula of (OD_sample_—OD_control_)/OD_control_. Conditioned media of cell cultures were also collected at the end of the experiment, and TNF-α concentration was estimated by ELISA according to the manufacturer’s protocol (Human TNF-alpha Quantikine ELISA Kit, R&D systems, Minneapolis, USA).

### Small interfering RNA transfection

The RA-FLS treated with IL-1β plus 5-AzadC were further transfected in 12-well plates (5×10^4^ cells/well) using Lipofectamine 2000 reagent (Invitrogen) according to the manufacturer’s protocol, with 100 nM (final concentration) mature miR-124a inhibitor (Ambion) or a scrambled control serving as a negative control (NC) (GenePharma Ltd., China). Each group had three wells. Cell growth was evaluated after 3 days and TNF-α concentration in the conditioned medium was estimated by ELISA as described previously.

### Statistical analysis

All the data were expressed as the mean ± standard deviation. The paired ***t***-test was used to evaluate the differences between groups. A *p* value less than 0.05 was considered as a significant difference.

## Results

### 5-AzadC triggered increase in the expression of miR-124a

**Fig 1:**
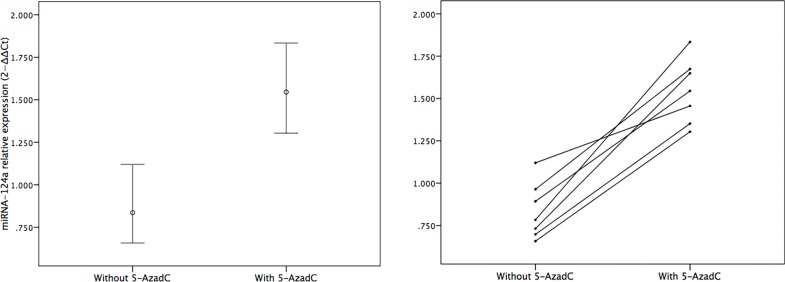
Analysis of miR-124a expression. FLS were isolated from 7 RA patients, cultured *in vitro*, and treated with 5-AzadC for 3 days. Expression of miR-124a in the treated cells was analyzed by real time quantitative PCR and represented either on the average (left) or individually (right).

FLS were derived from seven RA patients separately, cultured *in vitro*, and treated with 5-AzadC for 3 days. Expression of miR-124a mRNA was determined by real time quantitative PCR in the FLS samples with or without 5-AzadC treatment. As shown in Fig 1, the relative miR-124a expression in FLS without 5-AzadC treatment was about 0.836 ± 0.166, while that in the cells treated with 5-AzadC was about 1.545 ± 0.189, which is significantly higher than the former (*p*< 0.001).

### 5-AzadC caused demethylation of miR-124a genes

Three genomic loci (miR-124a-1 at 8p23.1, miR-124a-2 at 8q12.3, and miR-124a-3 at 20q13.33) are known to encode the same mature form of miR-124a [5]. The methylation status of these three miR-124a genes in FLS with or without 5-AzadC treatment was evaluated by MSP. The proportion of methylation at these three loci was 66.7% (14/21) in FLS without 5-AzadC treatment (Table 1), whereas proportion in FLS treated with 5-AzadC decreased to 38% (8/21), significantly less than the former (|^2^ = 4.19, *p* = 0.041).

**Table 1:** Frequency of DNA methylation at three miR-124a genes.

	Before 5-AzadC		After 5-AzadC		*P* value
	M	U	M	U	
miR-124a1	5	2	3	4	
miR-124a2	5	1	2	5	
miR-124a3	4	3	3	4	
Total	14	6	8	13	0.041

Note: M, methylated; U, unmethylated.

### 5-AzadC reduced IL-1β-mediated cell proliferation and TNF-α expression

IL-1β increased the proliferation of RA-FLS in a dose-dependent manner (from 0.1-10ng/ml) over time (data not shown). In our experiment, 5ng/ml IL-1β was used to treat the cells. Also we have tested different concentrations of 5-AzadC: 0.1μM, 0.5μM, 1μM and found that under 1μM, the cell proliferation was affected, but cell growth was not inhibited. Thus 1μM5-AzadC was used.

**Fig 2:**
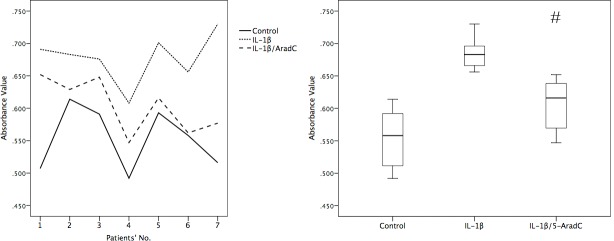
5-AzadC reduced IL-1β-mediated cell proliferation. FLS were treated with 5 ng/mL IL-1β alone for 4 days, or with IL-1β for 24 h and then with 1 μM 5-AzadC for 3 days, or without any treatments (control). Cell proliferation was estimated as described in the Materials and Methods. Cell growth was represented either individually (left) or on the average(right). ^#^, comparison with the IL-1β group, *p*< 0.05.

After incubation in the media supplemented with IL-1β for 4 days, the number of RA-FLS was apparently increased by 22.6% in average comparing with the cells grown in the absence of IL-1β. However, adding 1 μM 5-AzadC to RA-FLS 24 h after IL-1β stimulation caused only 9.22% increase in average in cell growth, which was significantly lower than that without adding 5-AzadC (*p* = 0.006) (Fig 2).

**Fig 3:**
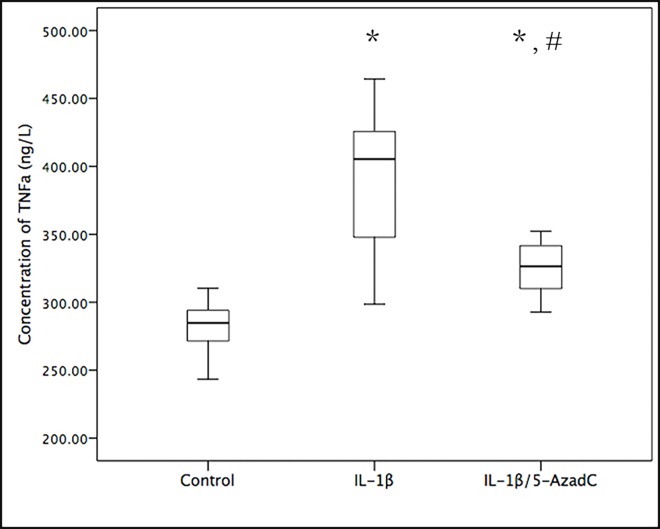
Analysis of TNF-α expression. FLS were treated exactly as described in the legend for Fig 2. The conditioned media were collected from each group, and the concentration of TNF-α in the conditioned media was estimated by ELISA. *, comparison with the control group, *p*<0.05; ^#^, comparison with the IL-1β group, *p*< 0.05.

We also measured TNF-α concentrations in the conditioned media of RA-FLS. As shown in Fig 3, the average TNF-α concentration in IL-1β group was significantly higher than that in the control group (387.91 ± 58.51 ng/L vs 281.35 ± 21.89 ng/L, p = 0.001). In contrast, the average TNF-α concentration in the IL-1β/5-AzadC group showed only a modest increase (324.99 ± 22.73ng/L), which was significantly less than that in the IL-1β group (p = 0.022).

### Transfection of miR-124a inhibitor increased cell proliferation and TNF-α expression

In RA-FLS transfected with miR-124a inhibitor, the average OD_450_ was 0.735±0.041 and the average TNF-α concentration was 423.78 ± 43.12 ng/L, which was significantly higher than those in FLS transfected with scrambled control (OD_450_ was 0.622 ± 0.029 and TNF-α concentration was 356.25 ± 50.82 ng/L, p = 0.001 and 0.011, respectively).

## Discussion

Abnormal hyperplasia of FLS contributes to RA progression and may be etiologically regulated by miRNAs and epigenetic changes such as DNA methylation [10] [11]. The methylome signature in RA-FLS also differs from that of osteoarthritis or normal FLS [12], suggesting the presence of a RA-specific epigenetic change. Yet, little is known about the genes that are specifically methylated in RA progression. We have been interested in miR-124a because it inhibits cell proliferation and is often silenced in a variety of diseased cells, including cancer cells and RA-FLS [6]. We have previously demonstrated that the promoter regions of miR-124a were hypermethylated [5]. After treating RA-FLS with 5-AzadC, a compound with the potency to inhibit the activity of DNA methyltransferase, thereby causing DNA demethylation and gene activation by opening chromatin for the access to transcription factors, the expression of miR-124a was significantly increased and MSP proved that miR-124a genes were demethylated. In some RA samples, methylation of miR-124a genes remained to be detected after 5-AzadC treatment, presumably due to variations in the demethylation imposed by 5-AzadC. These data showed that miR-124a gene methylation contributed in the transcriptional down-regulation of miR-124a in RA-FLS. However, this might not be the direct mechanism, as some research indicated histone modification also plays a role in it [13]. Thus, further experiments are needed to elucidate the exact pathway.

Furthermore, in the presence of 1 μM 5-AzadC, IL-1β induced significantly less degree of RA-FLS proliferation and less production of TNF-α than that in the absence of 5-AzadC. In order to address the pathological relationship between DNA methylation of miR-124a and RA-FLS proliferation as well as TNF-α expression, we used miR-124a inhibitor to transfect RA-FLS treated with IL-1β plus 5-AzadC. We found that after transfection, RA-FLS regained their proliferation ability and TNF-α expression was also increased. Overall, our data supported the view that up-regulation of miR-124a is associated with decreased RA-FLS proliferation and decreased TNF-α expression, which is consistent with the research of Yang S. et al [14].

5-AzadC as a demethylating agent has been recently used for treatment of myelodysplastic syndromes and acute myelomonocytic leukemia. Miao CG et al reported that 5-AzadC could increase the expression of secreted frizzled-related protein 4 in RA-FLS along with down-regulation of β-catenin and fibronectin [11]. 5-AzadC might be also used for treatment of patients with other types of chronic inflammatory disorders such as Sjögren's syndrome [15]. However, the mechanism for the 5-AzadC-associated anti-inflammatory activity has yet to be defined, and its application in rheumatic diseases remains largely unexplored. Thus, our finding that 5-AzadC reduces RA-FLS proliferation and TNF-α expression would not only provide an explanation for the action of 5-AzadC in the inhibition of inflammation but also imply a potential benefit of 5-AzadC for patients with RA.

## References

[1] Cooles FA, Isaacs JD. Pathophysiology of rheumatoid arthritis. Curr Opin Rheumatol. 2011; 23:233–40 10.1097/BOR.0b013e32834518a3 21427580

[2] Roberston KD. DNA methylation and human disease. Nat Rev Genet. 2005; 6:597–610 10.1038/nrg1655 16136652

[3] Ballestar E. Epigenetic alterations in autoimmune rheumatic disease. Nat Rev Rheumatol. 2011; 7:263–71 10.1038/nrrheum.2011.16 21343899

[4] Nakamachi Y, Kawano S, Takenokuchi M, Nishimura K, Sakai Y, Chin T, et al MicroRNA-124a is a key regulator of proliferation and monocyte chemoattractant protein 1 secretion in fibroblast-like synoviocytes from patients with rheumatoid arthritis. Arthritis Rheum. 2009; 60:1294–304 10.1002/art.24475 19404929

[5] Qiao Z, Li L, Guixiu S, Jing Z, Tong W, Bin Z. Research of the methylation status of miR-124a gene promoter among rheumatoid arthritis patients. Clin Dev Immunol. 2013 10 10 10.1155/2013/524204 24223605 PMC3810484

[6] Lujambio A, Ropero S, Ballestar E, Fraga MF, Cerrato C, Setien F, et al Genetic unmasking of an epigenetically silenced microRNA in human cancer cells. Cancer Res. 2007; 67:1424–9 10.1158/0008-5472.CAN-06-4218 17308079

[7] Nakano K, Whitaker JW, Boyle DL, Wang W, Firestein GS. DNA methylome signature in rheumatoid arthritis. Ann Rheum Dis. 2013; 72:110–7 10.1136/annrheumdis-2012-201526 22736089 PMC3549371

[8] Nervi C, De Marinis E, Codacci-Pisanelli G. Epigenetic treatment of solid tumours: a review of clinical trials. Clin Epigenetics. 2015; 7:127 10.1186/s13148-015-0157-2 26692909 PMC4676165

[9] Firestein GS. Invasive fibroblast-like synoviocytes in rheumatoid arthritis. Passive responders or transformed aggressors? Arthritis Rheum. 1996; 39:1781–90 8912499 10.1002/art.1780391103

[10] Arnett FC, Edworthy SM, Bloch DA, McShane DJ, Fries JF, Cooper NS, et al The American Rheumatism Association 1987 revised criteria for the classification of rheumatoid arthritis. Arthritis Rheum. 1988; 3:315–24 10.1002/art.1780310302 3358796

[11] Miao CG, Yang YY, He X, Xu T, Huang C, Huang Y, et al New advances of microRNAs in the pathogenesis of rheumatoid arthritis, with a focus on the crosstalk between DNA methylation and the microRNA machinery. Cell Signal. 2013; 25:1118–25 10.1016/j.cellsig.2013.01.024 23385088

[12] Nakano K, Boyle DL, Firestein GS. Regulation of DNA methylation in rheumatoid arthritis synoviocytes. J Immunol. 2013; 190:1297–303 10.4049/jimmunol.1202572 23277489 PMC3552038

[13] Chen XY, He DD, Xiang DD, Feng D, Wang J, Wang LH, et al MicroRNA-124a Is Epigenetically Regulated and Acts as aTumor Suppressor by Controlling Multiple Targets inUveal Melanoma. Invest Ophthalmol Vis Sci. 2013;54:2048–56 10.1167/iovs.12-10977 23404119

[14] Yang S, Qi L, Huan G, Dong PX, Yi LY, Ding FS, et al Micro RNA-124 mediates the cholinergic anti-inflammatory action through inhibiting the production of pro-inflammatory cytokines. Cell Research. 2013; 23:1270–1283 10.1038/cr.2013.116 23979021 PMC3817544

[15] Motegi K, Azuma M, Tamatani T, Ashida Y, Sato M. Expression of aquaporin-5 in and fluid secretion from immortalized human salivary gland ductal cells by treatment with 5-aza-2'-deoxycytidine: a possibility for improvement of xerostomia in patients with Sjogren's syndrome. Lab Invest. 2005; 85:342–53 10.1038/labinvest.3700234 15640830

